# Self-performed transnasal esophagogastroduodenoscopy in the standing position

**DOI:** 10.1016/j.igie.2025.04.004

**Published:** 2025-05-07

**Authors:** Shigenori Masaki, Chizuko Yamada

**Affiliations:** 1 Department of Surgery and Gastroenterology, Miyanomori Memorial Hospital, Sapporo, Hokkaido, Japan; 2 Department of Nursing and Safety Management, Miyanomori Memorial Hospital, Sapporo, Hokkaido, Japan

Esophagogastroduodenoscopy (EGD) is typically performed by endoscopists on patients, but reports indicate that endoscopists can also perform EGD on themselves.^1^ However, the optimal approach for self-performed EGD remains unclear. Here, we describe a method for self-performed transnasal EGD in the standing position and assess its feasibility and reliability.

Preparation included oral dimethylpolysiloxane (5 mL, 100 mg), intranasal naphazoline spray, and nasal anesthesia with 5 mL 2% lidocaine jelly. Ten minutes after preparation began, the endoscopist stood facing the monitor, held the ultrathin endoscope (Fujifilm, EG-L580NW7, Tokyo, Japan), and initiated the EGD (Fig. 1A), assisted and supervised by a nurse and a clinical engineer.

**Figure 1:**
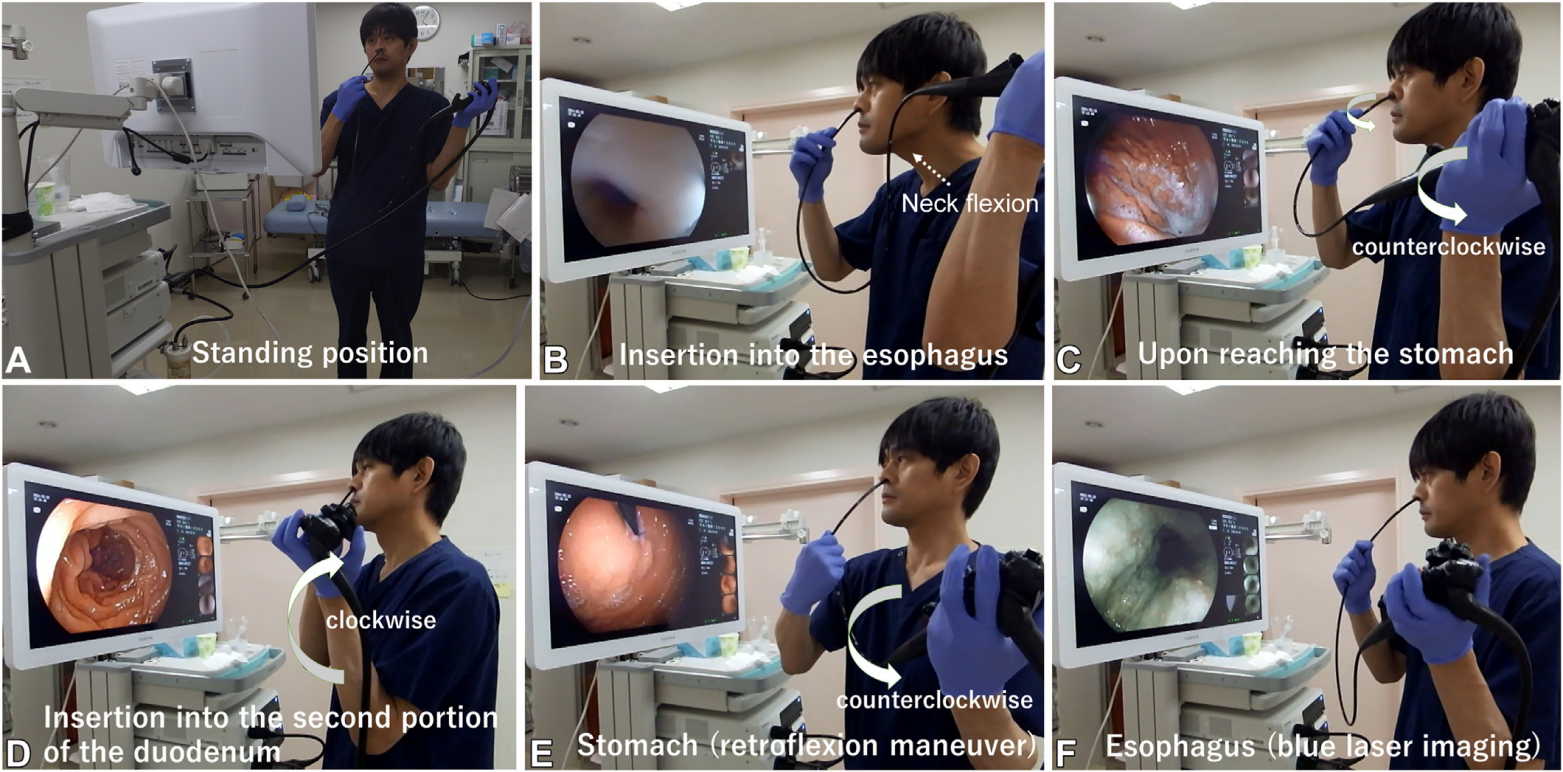
**A,** Procedure for self-performed transnasal esophagogastroduodenoscopy in the standing position (demonstrated by S. Masaki). **B,***Dotted arrow* indicates the direction of neck flexion to facilitate passage of gastroscope into esophagus seen on the monitor. **C-E,***Thick rotational arrow* indicates the direction of endoscope rotation. **C,** Upon reaching the stomach, counterclockwise torque with the left arm was needed to reach duodenum. **D,** To enter the second portion of the duodenum from the bulb, clockwise torque with the left arm was needed. **E,** Retroflexion in the stomach was possible with counterclockwise torque. **F,** Blue-laser imaging used to assess esophagus.

The endoscope passed smoothly through the nasopharynx into the esophagus. Successful esophageal insertion involved advancing the endoscope without manipulating the control knobs while deeply swallowing and flexing the neck forward (Fig. 1B). Upon the endoscope reaching the stomach, counterclockwise left-arm torque enabled the endoscope to reach the duodenum (Fig. 1C). After observation of the duodenal bulb, the endoscope was gently advanced with upward tip deflection and clockwise left-arm rotation to enter the second portion of the duodenum (Fig. 1D). The endoscope was then retracted into the stomach, where the entire gastric mucosa was examined by use of standard techniques, including retroflexion (Fig. 1E). Finally, the esophagus and pharynx were evaluated with blue-laser imaging during endoscope withdrawal (Fig.1F, Video 1, available online at www.igiejournal.org.).

The transnasal endoscope is preferable for self-performed EGD because its smaller diameter minimizes the gag reflex and facilitates the unsedated procedure.^2^ Technological advancements have made its image quality and field of view comparable with those of conventional transoral endoscopy for screening.^3^ The standing position allows unrestricted movement of the endoscopist’s left arm and upper body, and it offers better monitor visualization. By contrast, the seated or left-lateral position restricts this mobility, making the procedure significantly more difficult. Therefore, we consider the standing position optimal. Indeed, we have performed this method >10 times over the past 20 years without adverse events. However, monitoring is essential, and the procedure should be immediately discontinued if any concerning symptoms, such as vasovagal responses, occur.

Self-performed EGD offers 2 key benefits: enhancing endoscopic skills by gaining deeper insight into the patient’s experience during EGD, especially for trainees,^4^ and enabling endoscopists to monitor their own gastrointestinal (GI) health. In conclusion, self-performed transnasal EGD in the standing position appears to be a feasible and reliable approach for GI endoscopists to develop procedural expertise and to support GI self-care.

## Patient Consent

Written informed consent was obtained from the subject (author) for the publication of their information and imaging.

## Disclosure

All authors disclosed no financial relationships.

## Supplementary data

Video 1 Self-performed transnasal esophagogastroduodenoscopy procedure in the standing position. The pharynx and upper GI tract were observed smoothly without adverse events.

## References

[1] Horiuchi A., Nakayama Y. (2003). Small-diameter endoscope enabled endoscopist to detect his own duodenal erosion. Am J Gastroenterol.

[2] Dean R., Dua K., Massey B. (1996). A comparative study of unsedated transnasal esophagogastroduodenoscopy and conventional EGD. Gastrointest Endosc.

[3] Grant R.K., Brindle W.M., Robertson A.R. (2022). Unsedated transnasal endoscopy: a safe, well-tolerated and accurate alternative to standard diagnostic peroral endoscopy. Dig Dis Sci.

[4] Hu C.-T. (2010). Nasal anesthesia and body position changes: lessons learned from self-performed, transnasal EGD. Gastrointest Endosc.

